# Finite element modelling of atomic force microscopy imaging on deformable surfaces

**DOI:** 10.1039/d4sm01084a

**Published:** 2024-11-15

**Authors:** Joshua Giblin-Burnham, Yousef Javanmardi, Emad Moeendarbary, Bart W. Hoogenboom

**Affiliations:** a Department of Engineering Science, University of Oxford Wellington Square Oxford OX1 2JD UK joshua.giblin-burnham@linacre.ox.ac.uk; b London Centre for Nanotechnology, University College London 17-19 Gordon Street London WC1H 0AH UK b.hoogenboom@ucl.ac.uk; c Department of Physics and Astronomy, University College London Gower Street London WC1E 6BT UK; d Department of Mechanical Engineering, University College London Gower Street London WC1E 6BT UK

## Abstract

Atomic force microscopy (AFM) provides a three-dimensional topographic representation of a sample surface, at nanometre resolution. Computational simulations can aid the interpretation of such representations, but have mostly been limited to cases where both the AFM probe and the sample are hard and not compressible. In many applications, however, the sample is soft and therefore deformed due to the force exerted by the AFM tip. Here we use finite element modelling (FEM) to study how the measured AFM topography relates to the surface structures of soft and compressible materials. Consistent with previous analytical studies, the measured elastic modulus in AFM is generally found to deviate from the elastic modulus of the sample material. By the analysis of simple surface geometries, the FEM modelling shows how measured mechanical and topographic features in AFM images depend on a combination of tip-sample geometry and indentation of the tip into the sample. Importantly for the interpretation of AFM data, nanoparticles may appear larger or smaller by a factor of two depending on tip size and indentation force; and a higher spatial resolution in AFM images does not necessarily coincide with a more accurate representation of the sample surface. These observations on simple surface geometries also extend to molecular-resolution AFM, as illustrated by comparing FEM results with experimental data acquired on DNA. Taken together, the FEM results provide a framework that aids the interpretation of surface topography and local mechanics as measured by AFM.

## Abstract 2

Finite element modelling provides predictions for the appearance of atomic force microscopy (AFM) images, considering how a sample surface may be compressed by the AFM tip, with samples ranging from simple geometries to individual biomolecules.

## Graphical abstract

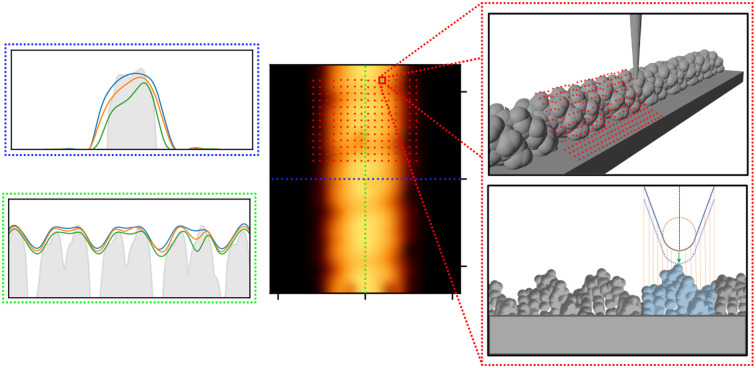

## 1 Introduction

Atomic force microscopy (AFM) is a versatile three-dimensional topographic technique implementing a mechanical probe to raster-scan and image sample surfaces. The technique provides reliable nanometer measurements of materials and has become a valuable tool with a diverse range of applications in areas such as materials physics, nanotechnology, electronics, and biology.^1–3^ In addition, AFM can image under natural conditions, such as in aqueous solutions and in real-time, allowing imaging of cell dynamics and biological processes. This includes imaging protein unfolding^4^ and conformational changes as a function of time,^5^ as well as mechanical characterization of soft and living materials.^6^

Along with these various strengths, AFM also comes with limitations. AFM-specific limitations are that the spatial resolution depends on the sharpness of the AFM tip, and that its operation requires a force between the tip and the sample:^7^ broadly speaking, sample features may appear broadened due to a convolution with the AFM tip, and even with an infinitely sharp tip, measured AFM topography may represent a deformed version of the unperturbed surface.

These limitations can in part be overcome using more advanced experimental methodology. For example, using principles borrowed from super-resolution optical microscopy, protruding parts of the samples surfaces may be displayed at a higher resolution than could be thought possible based on the finite width of the AFM tip.^8^ As for sample deformation, this can at least in part be accounted for by recording an unindented topography that can be derived from, *e.g.*, the (zero-force) contact point in force-*versus*-distance curves. While contact-point determination comes with its own subtleties,^9^ such an approach is facilitated by the routine integration of conventional and fast force spectroscopy methods in commercial AFM instrumentation, as well as bimodal operation,^10,11^ to yield estimates of the local surface indentation and thereby to allow for a reconstruction of the unindented surface.

Nonetheless, AFM remains a surface technique best suited for imaging protruding parts of a sample surface and it remains a technique that may underestimate the local sample height due to compression not accounted because of, *e.g.*, limitations in signal-to-noise-ratio of the tip-sample forces. It can therefore be beneficial to, alternatively, compare experimental results with computational simulations.^12^ For example, AFM images have been simulated by considering contours of closest mechanical contact between tip and sample, allowing predictions of how, *e.g.*, biological molecules may appear in AFM studies.^13–17^ Another approach consists of docking (approximately or fully) known biomolecular structures to their best fit within the surface envelope as measured by AFM,^18,19^ including best estimates for biomolecular orientation and tip shape,^20^ as well as flexible fitting *via* molecular-dynamics simulations.^21^

As early experimental studies, however, those computational approaches, broadly assume that AFM images represent an unindented surface topography. This assumption may be violated on soft matter, since the finite force applied by the AFM tip will generally result in elastic and/or viscous deformation of the surface. These effects have been extensively studied in the context of AFM-based force spectroscopy, an analysis aimed to discover local mechanical properties of a sample. By analytical modelling and/or computational simulations, force curves can be predicted and compared with the experimental data.^22–26^ A common approach in such computational simulations is finite element modelling (FEM). The FEM approach has been validated for AFM-based force spectroscopy^22^ and extended to also take into account viscoelastic effects in force curves.^27^

To predict how measured AFM topography is affected by mechanical deformation, we here apply such FEM simulations to AFM imaging. This also enables us to verify how local nanomechanical property measurement may depend on surface geometry and topography. Following a validation of our FEM implementation by comparison with analytical indentation models, we consider elastic globular and periodic surface topographies and explore how the measured surface morphology, the dimensions and the spatial resolution depend on the applied force that is inherent to most AFM imaging. Finally, we extend the observations on such general shapes to a direct comparison with experimental data on DNA, as a canonical biomolecule.

## 2 Methods

### 2.1 Tip geometry and analytical indentation models

**Fig. 1:**
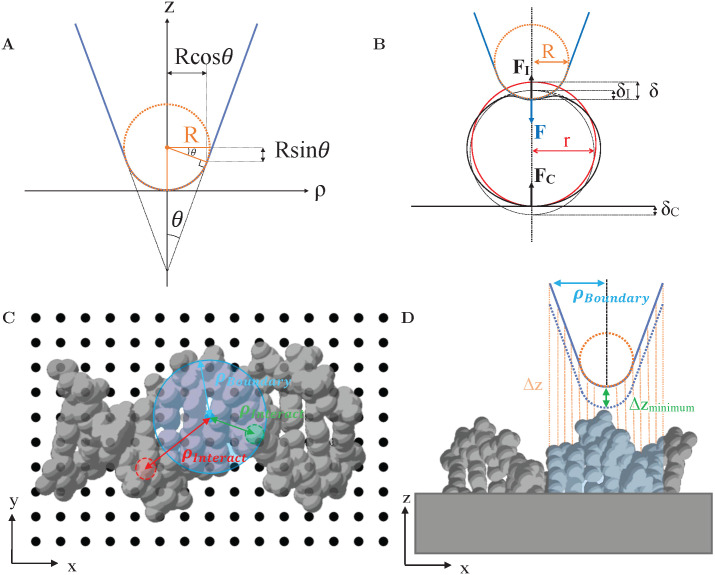
(A) Illustration of AFM tip geometry as a rigid cone with opening angle *θ* and with a spherical termination of radius *R*. (B) Schematic of an AFM tip indenting into a spherical sample of radius *r*. The applied force *F*_I_ results in the reaction forces *F* and *F*_C_ (C) Indication of different lateral (*x*, *y*) tip positions (black dots) with respect to a sample structure, here DNA (PDB: 2BNA^30^). Lateral distances between the tip position to constituent atoms are indicated by *ρ*_interact_. (D) Side view of (C), highlighting the calculation of vertical distances (Δ*z*) between the tip and the sample surface. To find the closest vertical distance (Δ*z*_min_) sample features were only considered when within a radius *ρ*_boundary_ (blue) defining the tip boundary with respect to the tip axis.

The tip geometry was specifically chosen to resemble the approximate shape of common AFM tips. As per the geometry of AFM tips in electron microscopy images,^28^ the AFM tip is here described as a rigid (incompressible) cone with opening angle *θ* = 20°, ending in a spherical termination of radius *R*,^29^ see Fig. 1A. The spherical portion smoothly transitions to the conical segment at the tangential contact point {*ρ*_t_, *z*_t_} = {*R *cos *θ*, *R*(1 − sin *θ*)}, where *ρ* is the radial coordinate away from the tip axis, and *z* the vertical coordinate measured from the tip end.

With this geometry, the tip can be considered spherical for indentations *δ* ≤ *R*(1 − sin *θ*) ≈ 0.65 *R*, which allows for a direct comparison of FEM simulations with analytical indentation models. The Hertz model describes the force *F* required for indenting a sphere of radius *R* into an elastic surface of radius *r* (with *δ* ≪ *R*, *r*), made of a homogeneous material with Youngs modulus *E* and Poissons ratio *ν*,^31–33^1
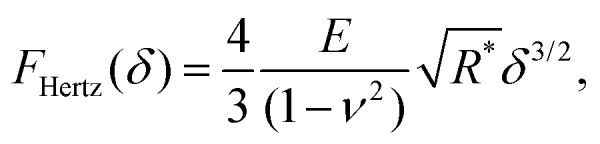
where the tip-surface contact radius R* is defined via R*^−1^ = *R*^−1^ + *r*^−1^. The Hertz model approximates spherical indenters by assuming a paraboloid shape,^34^ applicable for small indentations, which is the limit for which the analytical and FEM results will be compared hereafter.

Another commonly used description is the conical model provided by Sneddon, which presumes a perfectly conical tip of opening angle *θ*.^35^ For indentations into a sample surface with radius of curvature *r*, a modified form of Sneddon's model yields^23^2
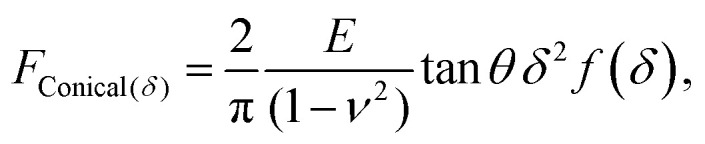
with3
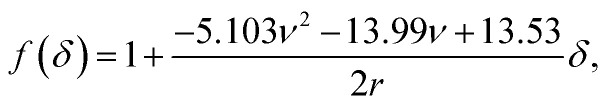
where the typical conical Sneddon model is modified by the experimentally fitted function *f*(*δ*) to produce closer force-indentation behaviour for spherical samples. By fitting measured force-*versus*-indentation curves with such models, it is possible to find estimates for the Young's modulus *E*.^25,36–39^

The models above ignore that there may be a compression at the contact between the soft sample and the hard substrate supporting the sample. However, the effects of the applied force *F* are generally distributed over the contact between the tip and the sample, with indentation *δ*_I_, and the contact between the soft sample and the underlying hard substrate, where the sample is indented and compressed by *δ*_C_,^40^ see Fig. 1B. AFM detects the applied force, *F*, which is equal to the reaction force at the tip-sample contact, *F*_I_, and the compressive reaction force at the sample-substrate contact, *F*_C_,

4 *F* = *F*_I_ = *F*_C_,

However, an AFM measurement detects the total displacement of the tip from first contact until an indentation *δ*, which is the sum of the local indentation at the top of the sample, *δ*_I_, and compression of the sample at the base, *δ*_C_,

5 *δ*(*F*) = *δ*_I_(*F*) + *δ*_C_(*F*),

which is larger than the indentation at the tip-sample contact.

Hertz and Sneddon indentation models (as in eqn (1)–(3)) assume the surface has infinite surface extent and, consequently, there is no compression at the base (*δ*_C_ = 0); they can therefore yield underestimates for the Young's modulus of the sample. By contrast, so-called “double contact” models^40,41^ do not neglect *δ*_C_ and the compressive effect. For a spherical indenter, the double-contact correction yields the following relation between measured force *F* = *F*_Double_ and measured indentation *δ*,^40,41^6
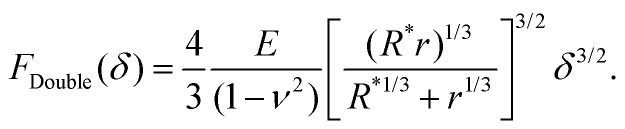


These analytical models can be used for a technical validation of the FEM implementation in our study. Given that in AFM experiments the contact radius is generally unknown, and considering the approximate validity of the Hertz model with R* ≈ *R* for *r* ≫ *R*, forces-indentation curves hereafter are expressed *via* the dimensionless force *F*/(E*R^2^) *versus* the dimensionless indentation *δ*/*R*, where E* = *E*(1 −*ν*^2^)^−1^. To facilitate comparison, the tip radius *R* of the Hertz and double contact models (eqn (1) and (6)) was also used to obtain dimensionless forces and indentations when applying the Sneddon model (eqn (2) and (3)).

### 2.2 Finite element modelling (FEM)

All FEM calculations were performed using the commercially available engineering software Abaqus (2017, Dassault Systèmes). Calculations used Abaqus's standard solver for quasi-static, implicit computations. Samples were modelled as continuous, homogeneous and isotropic elastic materials with Young's modulus, *E* = 100 MPa, and Poisson ratio, *ν* = 0.3 comparable to DNA and other biomolecules.^42–44^ The Young's modulus was further validated by fitting a Hertz model to the experimental data taken from Pyne *et al.*^45^ To eliminate the hourglass effect, R3D10 tetrahedral elements were employed.^46^ Simulations implemented “surface to surface contact” interaction with “hard”, nonadhesive normal contact and “rough” (Coulomb friction), non-slip tangential contact. Viscous effects were ignored throughout, for simplicity.

FEM was applied to various samples indented by AFM tips terminated with radius of curvature *R*. For comparison with the analytical indentation models indicated above, the tips were pressed against an elastic spheres of different radii *r*, resting on a rigid support, as in Fig. 1B, and cylindrical symmetry was imposed around the indentation (*z*) axis.

No such symmetry was imposed for FEM analysis of AFM imaging in which the lateral tip position was allowed to vary to determine AFM images. Consequently, a three-dimensional model of probe-sample indentation was required and the tip was modelled as a three-dimensional rigid conical surface with spherical termination. In that case, considering different tip radii *R*, the vertical (*z*) tip position was varied at different lateral (*x*, *y*) positions with respect to the sample structure, see Fig. 1C, and the resulting tip-sample forces determined by FEM. This procedure resulted in forces as a function of three-dimensional (*x*, *y*, *z*) tip position. AFM images or profiles where extracted by considering *xyz* contours of equal vertical force. The discrete *F*(*x*, *y*, *z*) data were interpolated using bi-cubic interpolation.

To reduce the number of FEM calculations, vertical (*z*) tip positions were limited to indentations *δ* ≥ 0, as forces were zero for other positions. To find these tip positions, the closest vertical distance between the tip and the sample was determined for each lateral (*x*, *y*) position of the tip, see Fig. 1D. This closest vertical distance was not necessarily at the end of the tip, but could also be where the sample touched the side of the tip. From there, the tip position was lowered, leading to indentations *δ* > 0 and non-zero forces. The calculation of closest vertical distances was limited to sample structures within a radius *ρ*_boundary_ set to define the largest radius with respect to the tip axis over which the tip could be expected to make contact with the sample (blue in Fig. 1C and D).

To test how indentation affected AFM images of biological molecules, biomolecular structures were imported from protein data bank (PDB) files and—again for simplicity—approximated as an elastic material with shape defined by the assembly of atoms and their van der Waals radii, see Fig. 1C and D for the example of DNA.

### 2.3 Analysis of periodic surface structures

To describe and quantify periodic variations of the AFM height *h*(*x*), a Fourier series was used,7
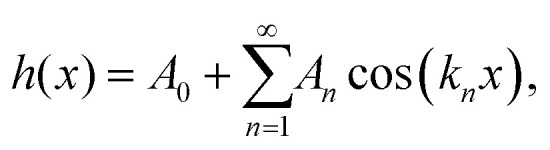
where *A*_*n*_ are the amplitudes of the different cosine functions characterized by wave numbers *k*_*n*_ = 2π/*λ*_*n*_ for variations with period *λ*_*n*_ = *λ*_1_/*n*. Given a choice of *x* = 0 for which *h*(*x*) is symmetric, the sine components of the Fourier series are zero.

## 3 Results

### 3.1 Validation of FEM against analytical indentation models

The FEM approach was first validated for AFM indentations into an elastic sphere, in which case analytical models are available (eqn (1)–(6)).

**Fig. 2:**
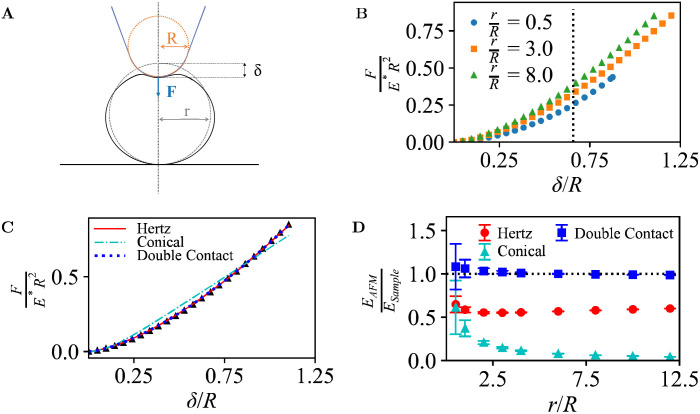
(A) Illustration of an AFM tip with radius *R* indenting into a spherical sample of radius *r*, mounted on a solid support, as in Fig. 1B. (B) Dimensionless force curves for indentations *δ*/*R*, for different *r*/*R*. For indentations with *r*/*R* ≤ 0.65 (dotted line), the contact area only extends over the spherical end of the tip, and the conical shape of the tip is irrelevant (see Section 2.1). (C) Data from B for *r*/*R* = 3.0, with indentation-model fits. (D) Measured Young's modulus *E*_AFM_ as determined from the fits, normalized to the Young's modulus of the sample *E*_Sample_, for different indentation models and ratios *r*/*R*.

Fig. 2B shows the extracted force-indentation data for various ratios *r*/*R* of the sphere radius *r* and the tip radius *R*, with forces and indentations given in dimensionless units, *F*/(E*R^2^) and *δ*/*R*, respectively (see Methods). When the commonly used Hertz model (eqn (1)) applies, this normalization should yield force-indentation curves that are independent of *R* as long as *r*/*R* ≫ 1. In that case *R* approaches the tip-contact surface radius R*, that is, the sample surface appears locally flat. Indeed, for the explored *r*/*R* range from 0.5 to 8.0, the curves are highly similar, with only a slight increase in dimensionless force for larger *r*/*R*. This increase is expected: when considering units in which the *R* dependence has been eliminated, as is the case here, a larger *r*/*R* implies that a larger fraction of the tip area is in contact with the sample, which results in a larger dimensionless indentation force for the same indentation.

The FEM simulations suggest a power-law scaling with the indentation. As common in AFM experiments, the force-indentation data can be fitted with Hertz or Conical indentation models (eqn (1)–(3) with tip-surface contact radius R*; see Fig. 2C),^36,37,39^ yielding estimates *E*_AFM_ for the Young's modulus of the sample *E*_Sample_.

However, these models do not account for the compression of the spheres at the base and, as a consequence, the fact that indentation at the tip-sample contact (*δ*_I_ in Methods) is smaller than the measured indentation in an AFM experiment *δ*. This effect is negligible in the limit *r* → ∞ but, otherwise, results in a tip-sample force *F* that appears to increase more slowly with the measured indentation *δ*, compared with *F versus δ*_I_. These effects can be accounted for by fitting the force-indentation (*F versus δ*) data directly with “double contact” models^40,41^ (eqn (6)).

Hence when comparing the fitted *E*_AFM_ with *E*_Sample_, one may expect a good match (*E*_AFM_/*E*_Sample_ ≈ 1) when applying the double-contact model. Without the double-contact correction, the expected *E*_AFM_ < *E*_Sample_, with potentially larger deviations for the Conical model since it assumes a (here erroneous) perfectly conical tip shape (see Methods). These expectations are met in Fig. 2D, confirming the validity of the FEM implementation.

### 3.2 AFM of elastic hemispheres

Next, to establish how AFM measures the topography and nanomechanics of simple geometries, an elastic hemisphere was probed. The hemisphere was modelled as a three-dimensional elastic body with radius *r* = 5 nm, attached to a fixed, rigid support at their base. This choice of geometry has two advantages compared with full sphere with a single contact point at the base as in Section 3.1. First, the force between the hemisphere and the support is spread over a larger area, such that the indentation at the bottom of the hemisphere is small compared with the indentation at the tip-sample contact, reducing the need for double-contact models to account for sample mechanics as in Section 3.1. Second, the wide base makes hemispheres less amenable to pivoting, rolling motion as readily occurs for off-axis indentation of perfect spheres.

**Fig. 3:**
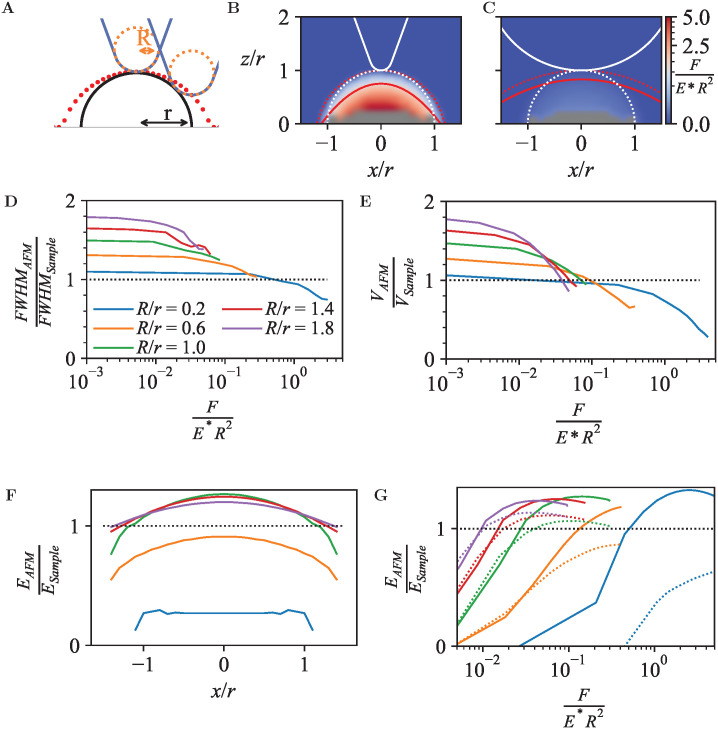
(A) Schematic of AFM probe (blue) with tip radius *R* (orange) tracing a hemispherical sample of radius *r* (black). Red dots indicate the trajectory of the tip end assuming no indentation. (B) Heat map showing the dimensionless indentation force for the geometry in A, with *R*/*r* = 0.2. Overlaid are the sample surface (white dotted line), the unindented AFM topography (red dotted line) and AFM topography at constant force *F*/(E*R^2^) = 0.23 (solid red line). Grey: No data available. (C) As B, for *R*/*r* = 1.4. (D) FWHM of the AFM topography (FWHM_AFM_), normalized to the FWHM of the hemisphere (FWHM_Sample_), as a function of *F*/(E*R^2^), for different *R*/*r*. (E) As D, for the apparent volume *V* of the hemisphere. (F) Measured Young's modulus based on Hertz model fitting as a function of scan position, for different (*R*/*r*) up to an indentation of *F*/(E*R^2^) = 0.1. (G) As F, for positions at the centre (solid) and at the edge of the hemisphere, *x*/*r* = 1 (dashed), as a function of *F*/(E*R^2^), for different *R*/*r*.

For any off-axis indentation, the first tip-sample contact will be at the side of the tip. Hence even for a rigid hemisphere, the trajectory of the tip end (red dots in Fig. 3A) will differ from the surface contour. This results in a wider appearance of the hemisphere in an AFM image, which is an effect well known as tip convolution.

In its simplest form (ignoring feedback errors and pecularities of dynamic modes particularly), the AFM topography does not represent the surface contour but contours of constant indentation force (*F*/(E*R^2^) in dimensionless units). As shown in Fig. 3B and C, deviations from the actual sample surface contours are due to both tip convolution and compression of the sample. To some extent, these effects compete, affecting the spatial resolution and volume as apparent in AFM images.

One way of defining spatial resolution is *via* the apparent widening of surface features in AFM images, considering the full-width half-maximum (FWHM)^47^ of a surface feature. Compared to the FWHM of the hemisphere, the measured FWHM is larger for low forces due to tip convolution, as has long been known and observed experimentally, *e.g.*, in nanoparticle size analysis.^48^ However, the FWHM can become smaller for higher forces (Fig. 3D). Hence by the application of higher forces, the apparent AFM resolution may increase, but this increase in resolution does not (necessarily) mean that the AFM measurement represents a more accurate representation of the surface topography.

Similar effects are observed for the apparent volume as would emerge from a grain-size analysis of AFM data, with deviations from the actual hemisphere volume that exceed ±50% in the data shown here (Fig. 3E). Experimentally, this affects volumetric measurements of protein complexes by AFM.^49,50^ Of note, there is less compaction of the hemispheres (at equal force) for larger tip radii *R*, since the indentation forces are spread over a larger area. Hence for robust size analysis of soft particles, blunter tips can yield more easily interpretable results, provided that the tip radius is known such that it can be corrected for (see, *e.g.*, ref. 45).

As in Section 3.1, the FEM data can also be fitted with indentation models, to extract apparent Young's moduli.^36,37,39^ Here, such apparent *E*_AFM_ are obtained from Hertz model fits (eqn (1)) to calculated force-indentation curves clipped to contours of equal indentation force. Consistent with the common approach in AFM experiments, where the curvature of the sample surface *r* is not generally known, R* is substituted by *R* for these fits.

This yields an effective Young's modulus, *E*_AFM_, as a function of relative lateral position, *x*/*r*, shown in Fig. 3F. At the centre (*x*/*r* = 0), the tip-sample contact area is largest and the normal force dominates. Hence the highest *E*_AFM_ is found there. For *R*/*r*≥1, this *E*_AFM_ is an overestimate (*E*_AFM_/*E*_Sample_ > 1) because of the solidity of the substrate that supports the hemisphere. Note the difference with the case in Fig. 2, where the Hertz model yields an underestimate of *E*_AFM_, attributed to compression at the contact of the sample with the solid support; for the hemisphere, this compression is much less relevant, as the force between hemisphere and substrate is spread over the entire hemisphere base and therefore leads to a much smaller compression at the base. This behaviour is not observed for the indentation where *R*/*r* < 1, conversely, *E*_AFM_ is underestimated at this low dimensionless force (*F*/(E*R^2^) = 0.23). Smaller indenters have smaller contact radius and, as such, experience a smaller elastic response. Consequently, the smaller indenters require smaller forces to achieve equivalent surface indentation.

*E*_AFM_ also depends on the depth of surface indentation, as shown in Fig. 2G. Based on fits up to given dimensionless forces *F*/(E*R^2^) in the FEM data, the central values for *E*_AFM_ peak at the same overestimated value at high forces. Beyond this force, the increase in indentation depth results in a slight reduction in effective Young's modulus, presumably related to a violation of the assumptions underpinning the Hertz model for deeper indentation *δ*.

However, for indentation at the side of the hemisphere (*e.g.*, at *x*/*R* = 1), higher forces lead to substantially reduced values of *E*_AFM_, which vary as a function of *R*/*r*, with smaller contact areas (for smaller *R*/*r*) leading to a softer response.

### 3.3 AFM of periodic surface structures

Besides single globular geometries as captured in Section 3.2, AFM images commonly include features that are at least locally periodic. This applies, for example, to atomic-resolution mapping of a solid–liquid interfaces,^51^ to two-dimensional lattices of membrane proteins,^52,53^ and to DNA imaging resolving the double helix.^45^ Generally, these samples may be considered as periodic soft materials, as here explored by considering a three-dimensional surface structure that is periodic in one dimension with a given wavelength *λ*, sinusoidally varying with amplitude *A*_Sample_ = *λ*. The structure has a width of 4*λ* and is attached to a solid support at its base. The solid support has a thickness *A*_Sample_ from the wave troughs down to its base. With this geometry, substrate and boundary effects may be considered small. The surface wavelength was set as *λ* = 10 nm and AFM tip radii were varied between 0.5–4.5 nm.

**Fig. 4:**
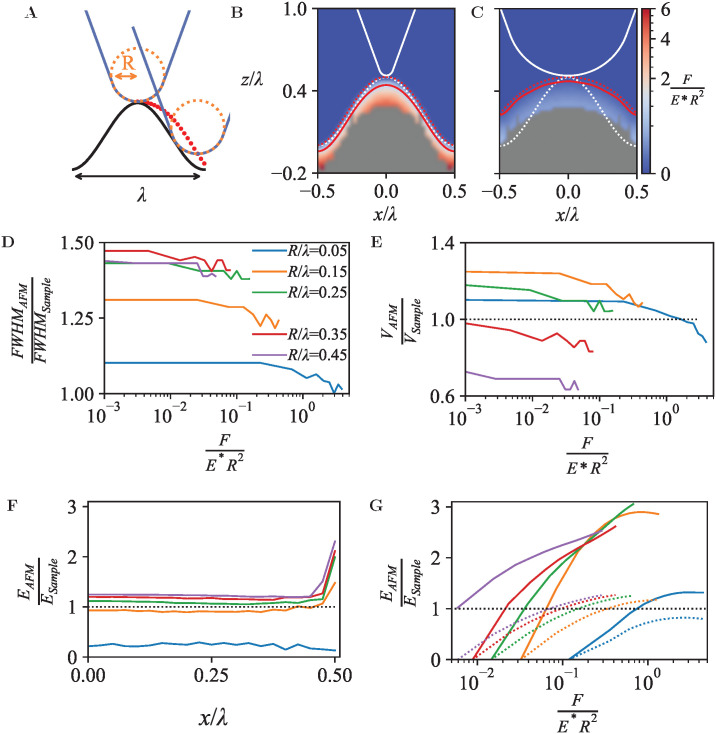
(A) Schematic of AFM probe (blue) with tip radius *R* (orange), shown at two different lateral (*x*) positions with respect to a sinusoidal surface structure (black) with periodicity *λ*. Red dots indicate the trajectory of the tip end assuming no indentation. (B) Heat map showing the dimensionless indentation force for the geometry in A, with *R*/*λ* = 0.05. Overlaid are the sample surface (white dotted line), the unindented AFM topography (red dotted line) and the AFM topography at constant force *F*/(E*R^2^) = 0.23 (solid red line). (C) As B, for *R*/*λ* = 0.45. (D) FWHM of the AFM topography (FWHM_AFM_), normalized to the FWHM of the hemisphere (FWHM_Sample_), as a function of *F*/(E*R^2^), for different *R*/*λ*. (E) As D, for the apparent relative volume *V* contained between the top of the crests and the bottom of the troughs in the AFM topography. (F) Measured Young's modulus based on Hertz model fitting, as a function of scan position, for different *R*/*λ* up to an indentation of *F*/(E*R^2^) = 0.2. (G) As F, for positions at the crest (dashed) and trough (solid lines), as a function of *F*/(E*R^2^), for different *R*/*λ*.

As is the case for any but the flattest surface geometries, the point of first tip-sample contact is often not at the very end of the tip, but at its side. This results in a broadening of the wave crest as measured by AFM, may reduce the trough depth, and moreover can lead to sharp drops in the AFM topography, following a trajectory that is locally steeper than the surface topography itself, see red dots in Fig. 4A.

As for the hemispheres in Fig. 3, these periodic structures can be indented with tips of different radii *R*, expressed in units of the surface periodicity *λ*, resulting in the force heat maps and contours of equal force in Fig. 4B and C. Plotted in Fig. 4D and E, the apparent FWHM and surface volume show a similar behaviour as for the hemispheres (*cf.*Fig. 3D and E), with features appearing broader and more voluminous at low forces, and instead appearing narrower and compacter due to the compression occurring at high forces.

In contrast to the behaviour observed for the hemispheres in Section 3.2, the effective Young's modulus *E*_AFM_ increases when the lateral position substantially deviates from the peak positions (crests) of the surface, shown in Fig. 4F and G. At the crests, the tip contacts the surface at the tip end only. At the surface troughs (*x*/*λ* = 0.5), however, the indenter experiences lateral contact from the surface on both sides, resulting in a larger tip-surface contact area and hence a larger effective Young's modulus than measured on the crests.

**Fig. 5:**
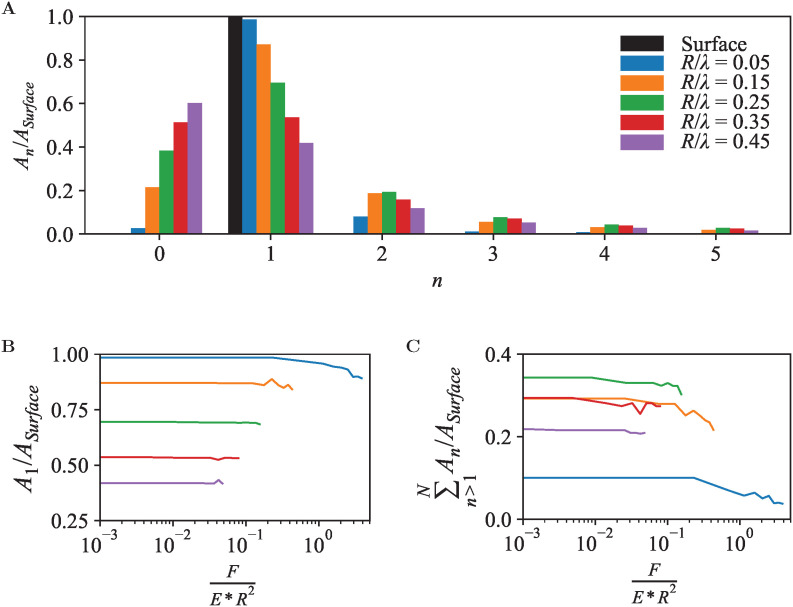
(A) Fourier series coefficients *A*_*n*_ for the contour of equal force *F*/(E*R^2^) = 0.227, for tip radii *R*/*λ*. (B) As A, but showing the variation of *A*_1_/*A*_Surface_ as a function of applied force *F*/(E*R^2^). (C) As B, for higher-order components 
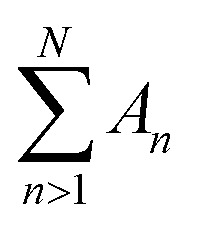
 where *N* = 50.

A periodic surface as in Fig. 4 also lends itself well for Fourier series analysis (eqn (7)), with amplitudes *A*_*n*≥1_ referring to oscillations with wave number *k*_*n*_ = 2π*n*/*λ* and *A*_0_ an offset, which in an AFM experiment depends on an arbitrary choice of reference height. For a periodic, perfectly (co)sinusoidal surface with wavelength *λ* as is the case here, the ideal AFM representation would yield *A*_1_ = *A*_surface_ and *A*_*n*>1_ = 0. This ideal scenario is closely followed for sharp tips (*R*/*λ* ≲ 0.1) at moderate load force (Fig. 5A and B).

For blunter tips (*R*/*λ* ≳ 0.1), however, the measured *A*_1_ drops noticeably below the amplitude of the surface wave, indicating that the AFM tip follows the surface contours less well and provides a diminished contrast. Higher-order Fourier coefficients *A*_*n*>1_ now significantly deviate from zero, due to the relatively steeper descend of the tip into the troughs of the wave. Hence if one uses Fourier analysis as is common in wave-based microscopies such as optical and electron microscopy, the AFM results would artefactually suggest the presence of features at a spatial resolution that is higher than possible given the structure of the actual surface.^54^

Again, these effects are dependent on the applied force (Fig. 5B and C). Broadly speaking, a higher indentation force results in a suppression of the amplitudes for the entire Fourier series. Not surprisingly, this indicates that as far as possible in the presence of noise, the best contrast and most accurate representation is obtained by a sharp tip and minimum indentation.

### 3.4 Comparison with experimental data on DNA

**Fig. 6:**
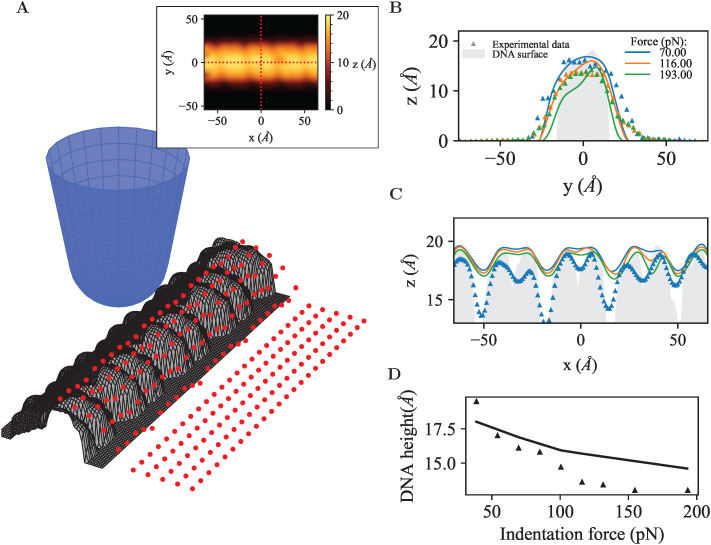
(A) 3D view of the simulated AFM tip probing a linear DNA segment, red points indicate initial scan positions across surface. Inset: Simulated AFM appearance of DNA molecule at indentation force *F* = 100 pN, dotted red lines indicate cross-section parallel to (*x* direction) and perpendicular to (*y* direction) the longitudinal axis of the molecule. (B) AFM topography measured across the DNA, comparing simulated data with experimental data. (C) AFM topography along the longitudinal axis of the DNA, showing the repeat of major and minor groves typical for the double helix. (D) DNA diameter as estimated from the maximum DNA height in AFM images across cross-section, as a function of applied force, comparing simulated data with experimental data. Experimental data was reproduced from Pyne *et al.*,^45^ denoted by the triangle symbols.

To explore how the effects above translate to less idealized geometries, the FEM approach was applied to DNA (Fig. 6A), as a canonical biological molecule for which high-quality experimental data are available in the literature. The starting point was the B-DNA structure (PDB: 2BNA),^30^ repeated to form a linearized DNA segment of 80 base pairs.^45^ With each atom represented as a sphere with radius defined by the atomic van der Waals radius from universal force field,^55^ a DNA FEM model resulted as a solid consisting of overlapping spheres, and mechanical properties defined by the shape of the model, and by the elastic modulus (set at 100 MPa) and Poisson ratio (set at 0.3) assumed as an effective value experienced by the AFM tip probing DNA on a solid support. Whereas molecular dynamics simulations may provide a better understanding of intramolecular detail, this FEM model facilitates the comparison with idealized geometries studied above and focuses on results that may more generally apply (*i.e.*, not defined by intramolecular dynamics). The tip radius was set to 18 Å based on experimental estimates in AFM experiments on DNA^45^ and a conical half-angle 5° following manufacturer's specifications.^56,57^ To mimic surface adhesion and simplify contact at the base, the molecule was partially embedded in a rigid base/substrate with the bottom 20% cleaved and fixed at the base using boundary conditions. The base itself was modelled as a rigid cube with width 50 Å and length 200 Å divided into bins of 5.5 Å, and in total 240 individual scan positions were used for the FEM simulations.

Firstly, tip convolution effects were clearly apparent in the cross-section of the DNA molecule (Fig. 6B) and *via* the smoothening of the repeating major and minor grooves typical for the DNA double helix (Fig. 6C). The measured compression and DNA width can be modelled to close agreement with the experimental data, as quantified by the profiles across the DNA molecule shown in Fig. 6B. However, in the simulations, the AFM tip does not seem able to penetrate as deep into the major and minor grooves as in the experimental data (Fig. 6C), which could be attributed to contrast arising through chemical differences in the local tip-sample interaction or to technical limitations of the model, such as an overestimate of the atomic radii or too coarse mesh sizes or tip positioning. Finally, the DNA height declines somewhat less rapidly in the simulations than in the experimental data, but captures the correct trend and order of magnitude, which is probably as good as may be expected without providing structural detail at atomic length scales.

Taken together, these data show that the continuum approach may be extended to single molecules and still provide qualitively correct results, while exact quantitativeness is hard to achieve without considering structural and possibly chemical details at atomic scales.

## 4 Discussion and conclusion

Unlike previous simulations of AFM images,^13–17^ the simulations presented here explicitly take into account the compression of soft materials that is inherent to most AFM measurements. By calculating the compressed surface topography of idealized geometries as well as a well-studied biological molecule (DNA), they provide a framework *via* which to assess the appearance of the sample surface in AFM experiments, both in terms of surface structure and surface mechanics.

Of direct relevance for many AFM applications, it is shown that sizes of nanoscale objects in AFM may be overestimated by tip broadening but also plausibly underestimated due to compression, by up to a factor 2 under the conditions investigated here. This provides indications for potential artefacts which AFM experimenters may wish to verify for, for example by independent assessment of tip size against a calibration sample and by verifying compression of the surface as a function of applied force. Moreover, it provides a quantification of how large such artefacts may be on various geometries, which may aid, for example, particle/molecular size analysis by AFM.

Similar caution applies to estimates of elastic moduli, which in in the simulations show larger than two-fold deviations from the true elastic modulus, depending on lateral position and applied force. Experimenters could address this by assessing position and force dependence of measured elastic moduli on samples with known elastic moduli. The here presented analysis provides a reference against which experimental behaviour can be compared.

As AFM experimenters continue to push for higher resolution, these simulations importantly show that the highest apparent resolution imaging does not necessarily result in the most accurate representation of the sample surface, which again calls for quantitative comparison against known structures.

Overall, the results illustrate how strongly the measured topography and nanomechanics may depend on tip and sample geometry and the applied forces in AFM experiments. As outlined in the Introduction, such dependency may in part be addressed by suitable experimental methodology, but direct comparison of AFM results and computational predictions is emerging as an potentially powerful additional approach.^12–21,58^ Here we have extend this by combining predictions for topographic imaging and mechanical indentation/characterization. We expect this to be a significant contribution towards more quantitative prediction-based interpretation of AFM images: hypotheses on sample/tip geometry and mechanics can be translated into testable predictions for experimental AFM results, and potentially be elaborated based on adaptive algorithms and/or machine-learning approaches informed by larger libraries of simulated AFM measurements.

One such structure is DNA, here simulated as a continuous solid, stretching the continuum approximation to the limit, still yielding qualitatively good comparisons with experimental data, but being less accurate in quantitative details. This highlights how mechanics could be included in AFM simulations based on structural information contained in, *e.g.*, the protein data bank, with the caveat that a finer FEM mesh size and thereby increased computational resources may be required for more quantitative results. This would be complementary to molecular dynamics simulations, which will be more accurate at atomic/submolecular length scales but harder to extend to mesoscale geometries.

Finally, we note that FEM simulations can be extended to also include viscous effects. While viscous effects may be negligible in some cases,^59^ they becomes more pronounced at higher load rates, as has been observed down to molecular length scales.^60^ This may result in smaller indentation depths, implying an underestimating of surface deformation and a misrepresentation of the true topography. Viscoelasticity may also cause overestimation of stiffness, as the material appears stiffer under faster loading conditions.^61^ While beyond the scope of the simulations presented here, this is a clear avenue along which it may be pursued to further enhance their predictive value.

Overall, We expect these computational approaches to find increasing use for the identification of measurement artefacts and for the quantitative interpretation of experimental data.

## Data availability

Source code, documentation, and related data are available at AFMsims repository at https://github.com/hoogenboom-lab/AFMsims. This includes python scripts for ABAQUS automation, and data analysis shown in article, alongside, indentation data collected from simulations.

## Conflicts of interest

B. W. H. holds an executive position at AFM manufacturer Nanosurf; Nanosurf played no role in the design and execution of this study. The other authors declare no conflicts of interest.
